# Emodin Ameliorates Acute Pancreatitis-Associated Lung Injury Through Inhibiting the Alveolar Macrophages Pyroptosis

**DOI:** 10.3389/fphar.2022.873053

**Published:** 2022-06-02

**Authors:** Xiajia Wu, Jiaqi Yao, Qian Hu, Hongxin Kang, Yifan Miao, Lv Zhu, Cong Li, Xianlin Zhao, Juan Li, Meihua Wan, Wenfu Tang

**Affiliations:** ^1^ Department of Integrated Traditional Chinese and Western Medicine, West China Hospital, Sichuan University, Chengdu, China; ^2^ Research Core Facility, West China Hospital, Sichuan University, Chengdu, China

**Keywords:** emodin, NLRP3 inflammasome, alveolar macrophage, pyroptosis, acute pancreatitis, acute lung injury

## Abstract

**Objective:** To investigate the protective effect of emodin in acute pancreatitis (AP)-associated lung injury and the underlying mechanisms.

**Methods:** NaT-AP model in rats was constructed using 3.5% sodium taurocholate, and CER+LPS-AP model in mice was constructed using caerulein combined with Lipopolysaccharide. Animals were divided randomly into four groups: sham, AP, Ac-YVAD-CMK (caspase-1 specific inhibitor, AYC), and emodin groups. AP-associated lung injury was assessed with H&E staining, inflammatory cytokine levels, and myeloperoxidase activity. Alveolar macrophages (AMs) pyroptosis was evaluated by flow cytometry. In bronchoalveolar lavage fluid, the levels of lactate dehydrogenase and inflammatory cytokines were measured by enzyme-linked immunosorbent assay. Pyroptosis-related protein expressions were detected by Western Blot.

**Results:** Emodin, similar to the positive control AYC, significantly alleviated pancreas and lung damage in rats and mice. Additionally, emodin mitigated the pyroptotic process of AMs by decreasing the level of inflammatory cytokines and lactate dehydrogenase. More importantly, the protein expressions of NLRP3, ASC, Caspase1 p10, GSDMD, and GSDMD-NT in AMs were significantly downregulated after emodin intervention.

**Conclusion:** Emodin has a therapeutic effect on AP-associated lung injury, which may result from the inhibition of NLRP3/Caspase1/GSDMD-mediated AMs pyroptosis signaling pathways.

## Introduction

Acute pancreatitis (AP) is one kind of inflammatory diseases of the pancreas with early local symptoms (e.g., pancreatic edema, abdominal pain, and distension *et al*) and followed by systemic inflammatory response (Xiao et al., 2016). Progression of AP into severe acute pancreatitis (SAP) usually leads to a life-threatening condition with multiple organ dysfunction, particularly acute lung injury (ALI), which is the cause of most deaths during the early phase of AP (Akbarshahi et al., 2012). Accumulating evidence has indicated that the overactivated immune system together with excessive inflammatory response plays a significant role in the disease progression (Zhang et al., 2016; Kumar, 2020). Therefore, how to effectively constrain local inflammatory response and attenuate AP-related lung injury (SAP-ALI) is key to improving the prognosis of AP and reducing mortality rate among early-stage AP patients.

Emodin is an anthraquinone compound, mainly derived from *rhubarb*, *Polygonum cuspidatum,* and other natural plants (Dong et al., 2016), which has been confirmed to possess anti-inflammatory bioactivity (Xue et al., 2015; Li et al., 2016). In recent years, numerous researches have reported the beneficial effects of emodin against AP progression *in vivo* and *in vitro* (Xu et al., 2016; Xia et al., 2019). Specifically, emodin was proved to ameliorate LPS-induced ALI by inhibiting the release of pulmonary inflammatory cytokines in rats and significantly reduce CIRP-activated inflammatory signaling pathways in rat alveolar macrophages (AMs) (Li et al., 2020; Xu et al., 2021). However, the effect and mechanism of emodin on SAP-ALI remain elusive.

Recently, The NOD-like receptor protein 3 (NLRP3) inflammasome, an intracytoplasmic multiprotein complex, containing NLRP3, apoptosis-associated speck-like protein (ASC) and procaspase-1, has been confirmed to participate in mediating the inflammatory damage process of ALI (Wu et al., 2013; Grailer et al., 2014). Activation of the NLRP3 inflammasome triggers caspase1 maturation and eventually induces caspase1-dependent pyroptosis, a new form of programmed cell death, characterized by pore-formation in the cell membrane, cell rupture, and the leakage of cytoplasmic contents (Shi et al., 2015). Moreover, a caspase-1 inhibitor Ac-YVAD-CMK (AYC) has been reported to alleviate LPS-induced lung injury by inhibiting caspase1-dependent AMs pyroptosis (Wu et al., 2015). AMs account for the major leukocyte population in the airways, through secreting a variety of inflammatory cytokines that can affect the development of ALI drastically under infectious or non-infectious conditions (Gordon et al., 2014; Murray et al., 2014). A growing body of evidence suggests that inflammation and cell death interplay with each other to create a cycle of auto-amplification that leads to further expansion of the inflammatory response. Extensive studies have demonstrated that AMs pyroptosis plays a critical role in acute lung inflammatory injury under various pathogenic conditions (e.g., endotoxin, mechanical ventilation, and *Streptococcus aureus*) (Wu et al., 2015; Yang et al., 2016; Yao and Sun, 2019). Notably, a recent study has demonstrated exosome-mediated activation of NLRP3 inflammasome and the consequent AMs pyroptosis might serve as a potential target for AP treatment (Wu et al., 2020). However, the effect of emodin on NLRP3 inflammasome-dependent AMs pyroptosis during SAP-ALI remains unclear.

To tackle this issue, we established two AP models of pancreatic ductal infusion of sodium taurocholate (NaT-AP) in rats and intraperitoneal injections with caerulein plus LPS (CER+LPS-AP) in mice and investigated the underlying mechanism of emodin on AP-related ALI.

## Materials and Methods

### Animals

Thirty-two male Sprague-Dawley rats (200–250g) and thirty-two C57BL/6J mice (22–25 g) were purchased from the Jiangan Laboratory Animal Center of the Sichuan University (Chengdu, China). All animals were housed in a controlled environment (temperature: 22 ± 2°C, humidity: 65 ± 10%) and received standard chow and water. Rats and mice received adapted feeding for 7 days and fasted for 24 h before AP induction. All the animal experiments were conducted in West China Science and Technology Park of Sichuan University and approved by the Experimental Animal Ethics Committee of the Sichuan University of West China Hospital (protocol number: 2020013A).

### Reagents and Antibodies

Sodium taurocholate (909688) was purchased from Bailingwei Technology Co., LTD. (Beijing, China) and emodin was obtained from Beijing Zhongke Quality Inspection (Beijing, China). Caerulein (C9026), LPS (L2880) and Ac-YVAD-CMK (SML0429) were purchased from Sigma-Aldrich (United States). Sodium pentobarbital (B5646) was purchased from Baoxin Biotechnology Co., LTD. (Chengdu, China). Anti-NLRP3 (ab263899), anti-GSDMD (ab219800) and anti-GSDMD-NT (ab215203) were obtained from Abcam (Milton, United Kingdom). Anti-ASC (DF-6304) and anti-Cleaved-Caspase 1 (p10, AF4022) were purchased from Affinity Biosciences (Jiangsu, China). Anti-GAPDH (10494-1-AP) was purchased from Proteintech Company (Chicago, United States).

### Establishment of Experimental SAP-ALI Models

Animals were randomly divided into four groups (*n* = 8 for each group): (1) sham group, (2) AP group, (3) AP + AYC group, and (4) AP + emodin group. NaT-AP was induced in rats as described in our previous work (Zhang et al., 2017). In brief, rats were anesthetized by intraperitoneally (i.p.) injecting 2% sodium pentobarbital (40 mg/kg.BW), then 3.5% sodium taurocholate (1 ml/kg) was retrogradely injected into the biliopancreatic duct of rats using a micro-perfusion pump at a rate of 0.1 ml/min. CER+LPS-AP mice were i.p. injected with caerulein 6 times hourly (50 μg/kg) followed by LPS (10 mg/kg) challenge immediately after the last caerulein injection (Ding et al., 2003). Controls were injected with an equal amount of saline. AYC (5 mg/kg), as a positive control, was i.p. injected 1 h before modeling and emodin (10 mg/kg) was intragastrically administered twice at 6 and 12 h post modeling. Our previous work showed that the high dose of emodin (10 mg/kg) was more effective than the low dose of emodin (5 mg/kg) for alleviating SAP-ALI. Therefore, 10 mg/kg of emodin was selected in our experiments (Hu et al., 2021).

Animals were anesthetized with i.p. injection of 2% sodium pentobarbital (40 mg/kg.BW) the next day (24 h after AP induction), blood was collected by cardiac puncture for the measurement of serum amylase and lipase activity. Lung tissue samples were collected to analyze the myeloperoxidase (MPO) activity and inflammatory cytokines. Pancreas and lung tissue samples were fixed for histological analysis. Bronchoalveolar lavage fluid (BALF) was extracted for AMs separation, and the proinflammatory cytokines and lactic dehydrogenase (LDH) released in the supernatant of BALF were evaluated by ELISA, then the collected AMs were subjected to western blot and flow cytometry.

### Detection of Inflammatory Factors in Lung Tissues Tables

After BALF collection, lung homogenates were prepared by the following methods. Briefly, lung tissues were added with 1 ml RIPA lysis buffer (Beyotime, China), then homogenized with a high-flux tissue homogenizer (MX-S, SCILOGEX, United States). After homogenization, the mixer was incubated at 4°C for half an hour and then centrifugated at 13000 ×*g* for 15 min. Finally, the collected supernatants were used to measure inflammatory cytokine levels (including IL-1β, IL-18, and TNF-α) by corresponding ELISA kits (Neobioscience, Shenzhen, China) following the manufacturer’s protocol.

### Lung MPO Activity

To assess the degree of infiltrated neutrophils, myeloperoxidase (MPO) activity in lung samples was measured by the MPO activity assay kit (Nanjing Jiancheng Bioengineering Institute, China), following the manufacturer’s instructions.

### Collection of Bronchoalveolar Lavage Fluid (BALF)

Intratracheal injection of 10 ml (1 ml for mice) phosphate-buffered saline (PBS), which also means bronchoalveolar lavage, was performed 4 times on the animals following the anesthesia. Then the collected BALF from the syringe was mixed and centrifugated at 250 × *g* for 5 min. IL-1β and IL-18 in the supernatant were measured using the corresponding ELISA kit (Neobioscience, China), and the level of lactate dehydrogenase (LDH) was measured by an LDH Assay Kit (Beyotime Biotechnology, Haimen city, China). Besides, the pelleted cells were resuspended in conditioned RPMI 1640 medium (fetal bovine serum: 10%; penicillin and streptomycin: 1%). Then they were incubated in 60-mm sterilized dishes (37°C; 5% CO2) for 2 h and then washed twice with a warm medium to remove nonadherent cells. Finally, the whole cells were harvested and subjected to western blotting.

### Western Blot

Collected AMs were resuspended on ice with RIPA lysis buffer containing proteinase inhibitors, phosphatase inhibitors, and PMSF for 30 min, then the supernatants were obtained after centrifugation for 15 min (12,000 × *g*, 4°C). Quantification of protein concentrations was determined by a bicinchoninic acid protein assay kit (Beyotime Biotechnology, Haimen city, China). Next, each lysate samples (25 µg/lane) were resolved on 12% SDS-PAGE gels and transferred onto nitrocellulose (NC) or polyvinylidene difluoride (PVDF) membranes. After being blocked in the blocking solution (EpiZyme Biotech, Shanghai, China) for 1 h, membranes were incubated with primary antibodies against NLRP3 (1:1000), Caspase 1 p10 (1:1000), ASC (1:200), GSDMD (1:1000), GSDMD-NT (1:1000), and GAPDH (1:2000) all night at 4°C. The next day, the membranes were washed with TBST 3 times and incubated with horseradish peroxidase (HRP)-labeled secondary antibody at room temperature for 1 h. Finally, blots were visualized through the Bio-Rad imaging system. The relative expression of pyroptosis-related proteins to GAPDH was analyzed and processed by ImageJ software.

### Determination of AMs Pyroptosis

The collected AMs from BALF were analyzed by flow cytometry to evaluate the ratio of pyroptotic cell death. Firstly, AMs were stained using FLICA® 660 Caspase-1 Assay regent (Immunochemistry Technologies, United States) at 37°C for 30min in the dark. Pelleted cells were washed two times and then stained with propidium iodide (PI) in the dark at room temperature for 5 min. Flow cytometry (Cytoflex, Beckman) analyzed the rates of double-positive staining (caspase-1 and PI) cells, which were also regarded to be pyroptotic cells.

### Histological Examination

The histopathological injury of pancreatic and lung tissues was evaluated after H&E staining. Two independent pathologists scored pancreatic injury by acinar cells edema, infiltrated inflammatory cells, focal hemorrhage, and necrosis (from 0 to 4) as described (Kusske et al., 1996). Assessment of pulmonary injury was scored by interstitial/intra-alveolar edema, alveolar wall thickening, inflammation, and hemorrhage (from 0 to 4) as described (Mikawa et al., 1994).

### Statistical Analysis

All Data were presented as mean (standard deviation) and analyzed using GraphPad Prism eight Software. Differences between multiple groups were evaluated by ANOVA and post hoc Tukey’s test. *p* < 0.05 was considered significantly different.

## Results

### Emodin Attenuated AP-Associated Lung Injury in NaT-AP Rats

**FIGURE 1:**
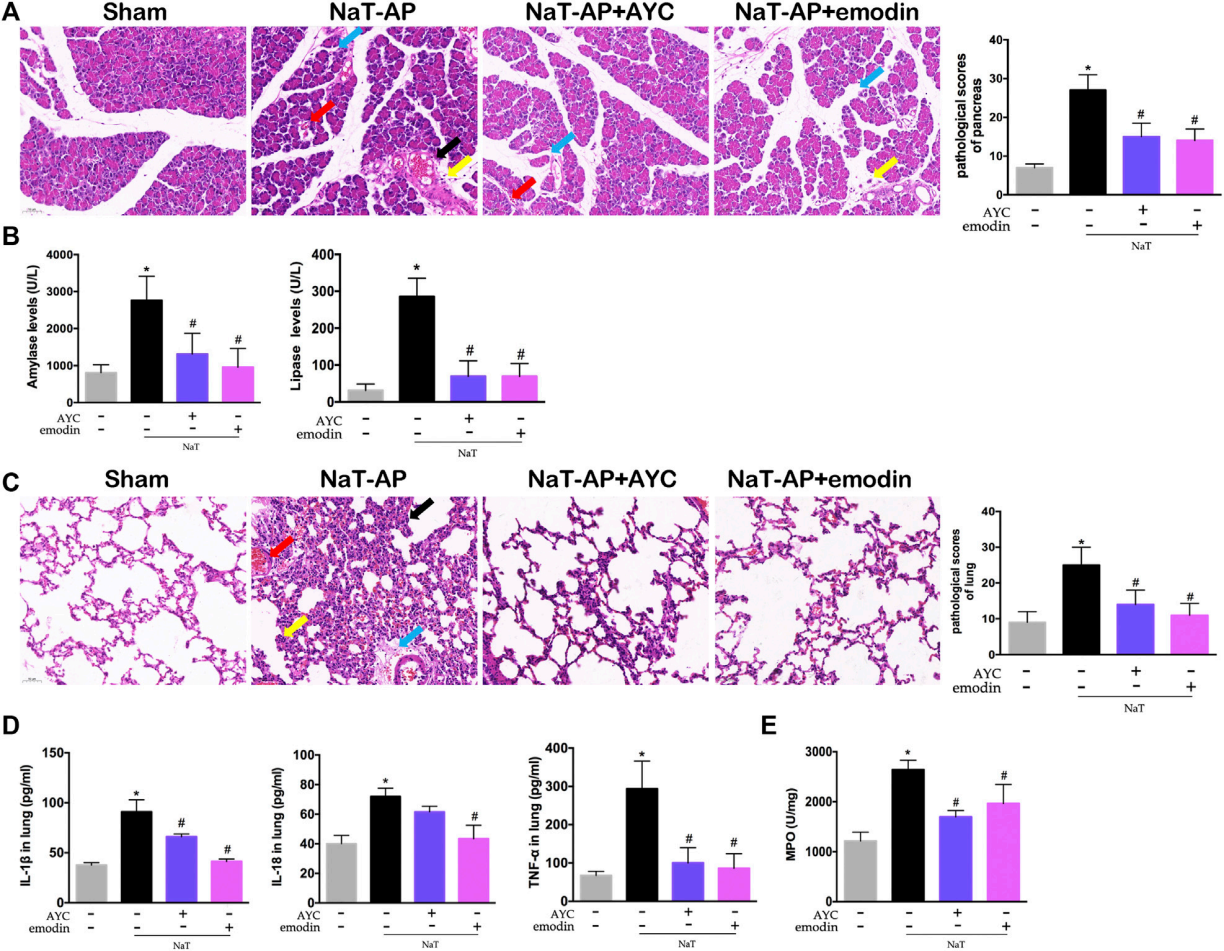
Emodin attenuated AP-associated lung injury in NaT-AP rats. **(A)** H&E staining images (×200) and pathological scores in pancreas from sham rats, NaT-AP rats, NaT-AP + AYC rats, and NaT-AP + emodin rats. Arrows (blue, yellow, red, black) indicated pancreatic edema, inflammation, hemorrhage, and necrosis respectively. **(B)** Serum amylase and lipase levels of each group. **(C)** H&E staining images (×200) and pathological scores in lung tissue of each group. Arrows (blue, yellow, red, black) indicated lung edema, hemorrhage, alveolar septal thickening, and inflammation respectively. **(D)** The levels of IL-1β, IL-18, and TNF-α within lung tissue of each group. **(E)** The expression of MPO activity within lung tissue of each group. ^*^ *p* < 0.05 vs. the controls. ^#^ *p* < 0.05 vs. NaT-AP rats.

In this study, two animal models (NaT-AP and CER+LPS-AP) were established. We first tested the effect of emodin on AP-associated lung damage in rats induced by 3.5% sodium taurocholate. As shown in Figure 1A, H&E staining results showed obvious acinar cells edema, infiltrated inflammatory cells, focal hemorrhage and necrosis in the pancreas of NaT-AP rats. Conversely, emodin treatment distinctly lowered the pathological injury of the pancreas, with reduced pathological scores. Serum amylase and lipase levels are commonly used biochemical markers in the diagnosis of pancreatitis (Rompianesi et al., 2017). The serum amylase and lipase levels of NaT-AP rats were markedly higher compared with those in the sham group, while they were downregulated by emodin administration. AYC exerted similar impact on serum amylase and lipase levels compared to emodin in rats (Figure 1B).

Then, the effects of emodin on pulmonary damage and inflammatory response were evaluated. NaT-AP rats showed significant pathological changes in the lung tissues, characterized by interstitial/intra-alveolar edema, hemorrhage, alveolar wall thickening and inflammation, accompanied by increased histopathological scores. After emodin and AYC administration, lung injury was effectively mitigated (Figure 1C). Moreover, the inflammatory mediators in lung tissue, including IL-1β, IL-18, and TNF-α significantly decreased after emodin and AYC intervention (Figure 1D). In addition, lung MPO activity in the AP group was enhanced, indicating extensive immersion of neutrophils, while the intervention of emodin and AYC both imposed inhibitory effect on neutrophil immersion in the lung tissue of AP rats (Figure 1E).

### Emodin Inhibited AMs Pyroptosis and Secretion of Inflammatory Factors in SAP-ALI Rats

**FIGURE 2:**
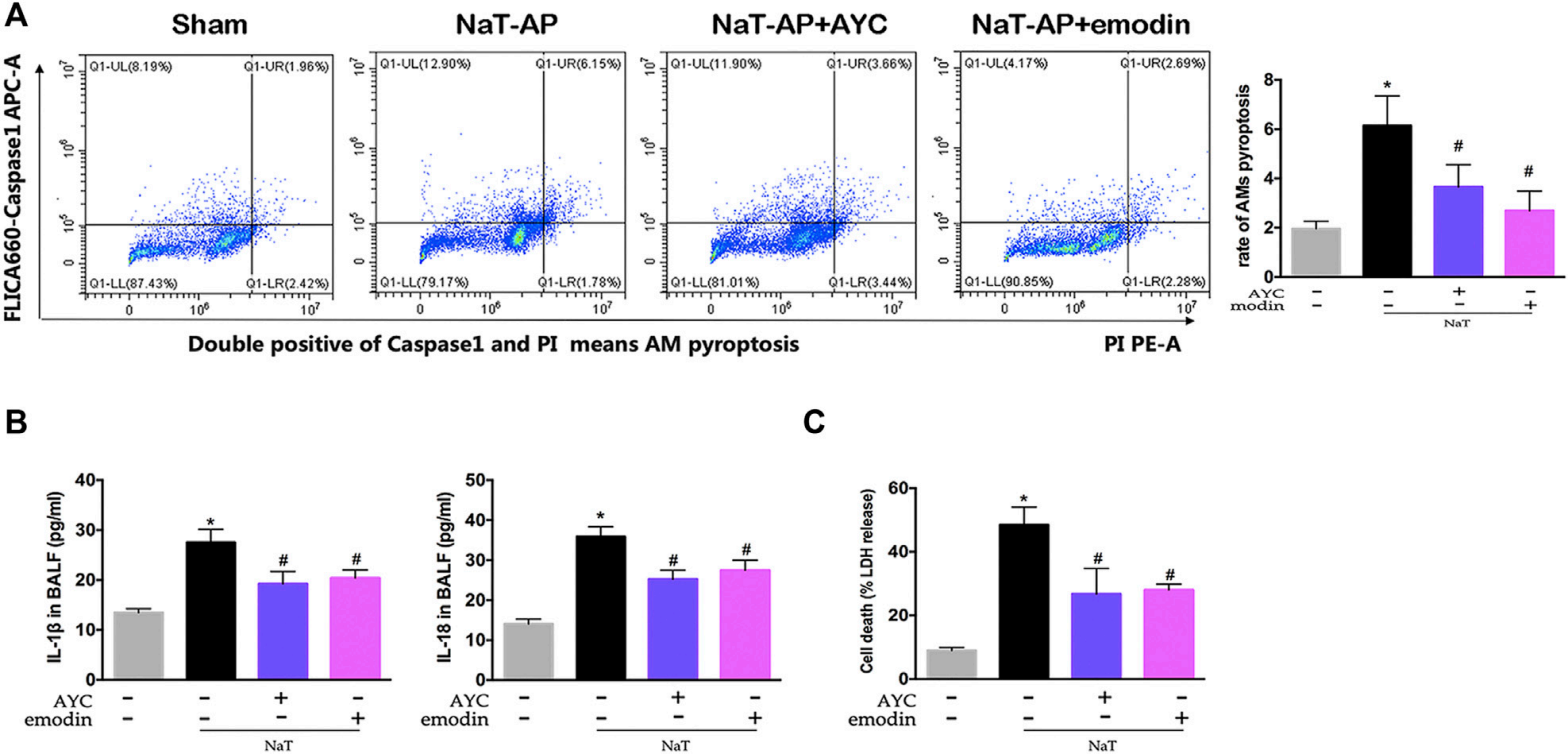
Emodin inhibited AMs pyroptosis and secretion of inflammatory factors in SAP-ALI rats. **(A)** The rate of pyroptotic AMs determined by flow cytometry from sham rats, NaT-AP rats, NaT-AP + AYC rats, and NaT-AP + emodin rats. Double-positive staining cells (caspase-1 and PI) were regarded to be pyroptotic cells. **(B)** the levels of IL-1β and IL-18 of each group in BALF supernatant. **(C)** The LDH level of each group in BALF supernatant. ^*^ *p* < 0.05 vs. the controls. ^#^ *p* < 0.05 vs. NaT-AP rats.

After collecting the BALF, the ratio of pyroptotic AMs was assayed by flow cytometry. NaT-AP group displayed a significantly higher frequency of caspase-1+PI AMs, a hallmark of cell pyroptosis, compared with other groups. Of note, emodin and AYC significantly prevented AMs pyroptosis in NaT-AP rats (Figure 2A). Furthermore, we quantified proinflammatory cytokines and analyzed the LDH level in BALF. As a result, IL-1β and IL-18 levels in BALF were much higher in AP rats compared with those in the controls, while these levels decreased after emodin and AYC treatment (Figure 2B). Typically, increased level of LDH released in BALF supernatant revealed a higher rate of cell damage or death. As a result, emodin and AYC treatment protected alveolar AMs by markedly reducing AP-induced LDH release in BALF in rats (Figure 2C).

### Emodin Inhibited AMs Pyroptosis by Targeting NLRP3-Caspase1-GSDMD Pathway in SAP-ALI Rats

**FIGURE 3:**
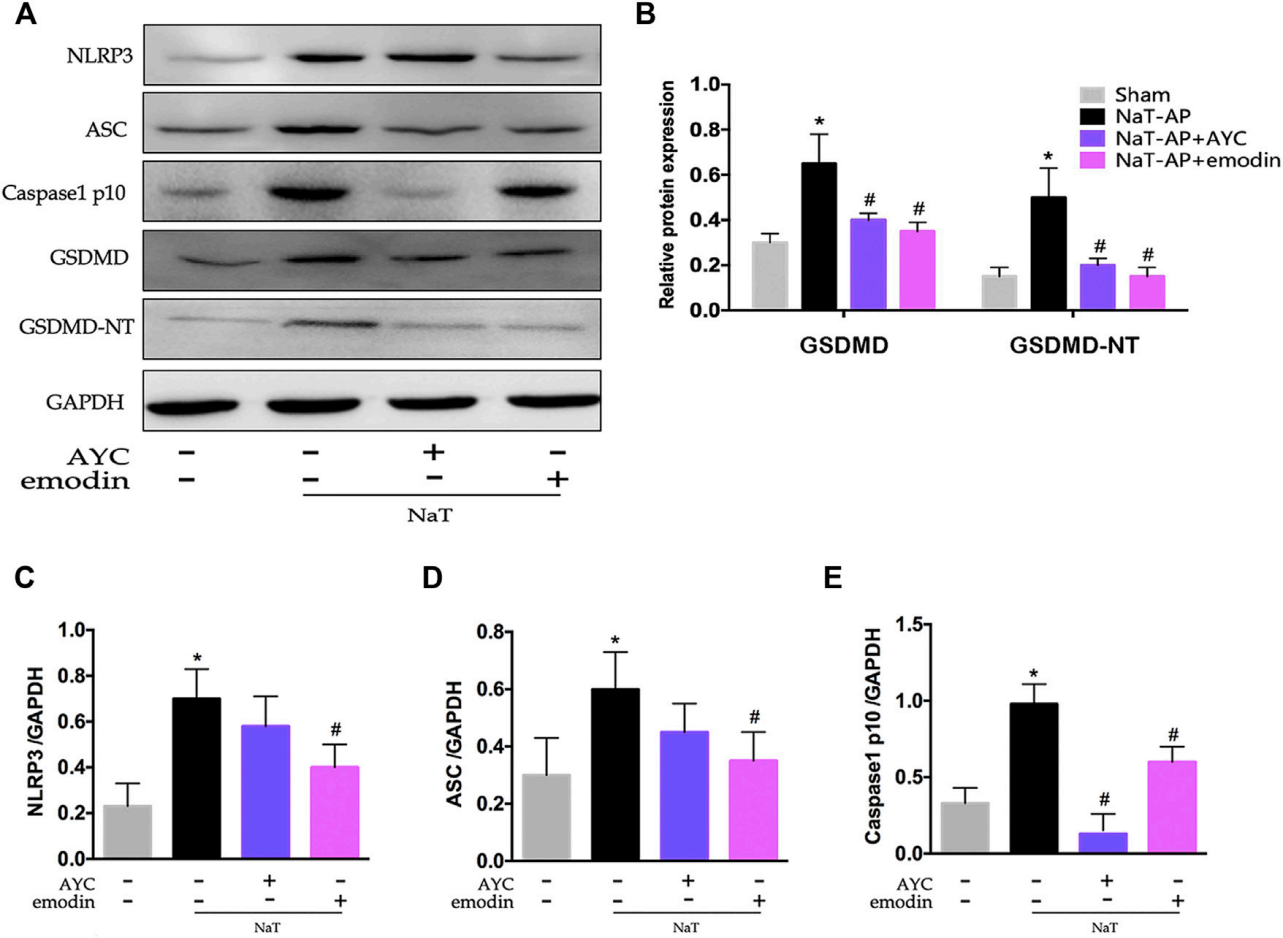
Emodin inhibited AMs pyroptosis by targeting NLRP3-Caspase1-GSDMD pathway in SAP-ALI rats. **(A–E)** Western blots and quantitative analysis of pyroptosis-related proteins (NLRP3, ASC, Caspase1 p10, GSDMD, and GSDMD-NT) relative to GAPDH from sham rats, NaT-AP rats, NaT-AP + AYC rats, and NaT-AP + emodin rats. ^*^ *p* < 0.05 vs. the controls. ^#^ *p* < 0.05 vs. NaT-AP rats.

Besides, we also examined the NLRP3 inflammasome and pyroptosis-related proteins by western blotting. Our results indicated that the protein expression levels of NLRP3, ASC, Caspase1 p10, GSDMD, GSDMD-NT in AMs were apparently elevated in NaT-AP rats, while all these markers mentioned above did not show obvious elevation in their expression levels when caspase1 blockade AYC came into play. Interestingly, emodin showed the same inhibitory effect on NLRP3-Caspase1-GSDMD pathway. The expressions of NLRP3, ASC, Caspase1 p10, GSDMD, GSDMD-NT were significantly decreased after emodin administration. (Figures 3A–E).

### Emodin Attenuated AP-Associated Lung Injury in CER+LPS-AP Mice

**FIGURE 4:**
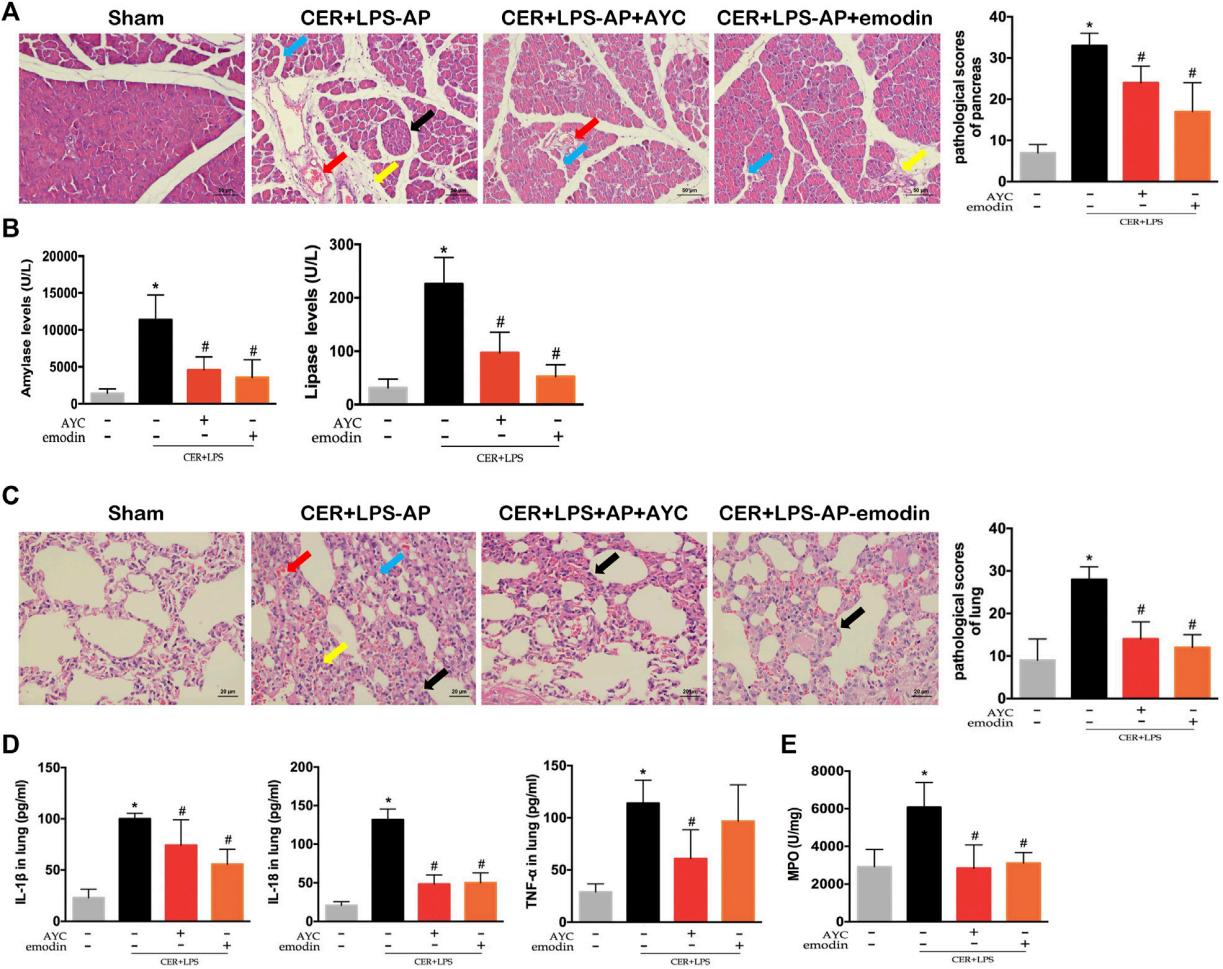
Emodin attenuated AP-associated lung injury in CER+LPS-AP mice. **(A)** H&E staining images (×200) and pathological scores in pancreas from sham mice, CER+LPS-AP mice, CER+LPS-AP + AYC mice, and CER+LPS-AP + emodin mice. Arrows (blue, yellow, red, black) indicated pancreatic edema, inflammation, hemorrhage, and necrosis respectively. **(B)** Serum amylase and lipase levels of each group. **(C)** H&E staining images (×200) and pathological scores in lung tissue of each group. Arrows (blue, yellow, red, black) indicated lung edema, hemorrhage, alveolar septal thickening, and inflammation respectively. **(D)** The levels of IL-1β, IL-18, and TNF-α within the lung tissue of each group. **(E)** The expression of MPO activity of each group. ^*^ *p* < 0.05 vs. the controls. ^#^ *p* < 0.05 vs. CER+LPS-AP mice.

After exploring the therapeutic effect and the underlying mechanism of emodin in NaT-AP rats, we as well investigated its influence on another AP model. H&E staining results showed obvious acinar cells edema, infiltrated inflammatory cells, focal hemorrhage and necrosis in the pancreas of CER+LPS-AP mice. Conversely, emodin treatment effectively alleviated pulmonary injury and lowered the corresponding pathological scores. Meanwhile, the levels of serum amylase and lipase were markedly increased in the CER+LPS-AP group while they were downregulated by either emodin or AYC administration, respectively (Figure 4B).

In the next step, pulmonary inflammatory damage was also evaluated in CER+LPS-AP mice. Significant pathological changes together with increased histopathological scores were observed in the lung tissue, consistent with those of NaT-AP rats. After either emodin or AYC administration, the lung injury was effectively ameliorated (Figure 4C). Moreover, the secretion of inflammatory cytokines including IL-1β, IL-18, and TNF-α in lung tissue exhibited a downward trend after emodin or AYC intervention (Figure 4D). In addition, MPO activity in the AP group was enhanced, while the intervention of emodin or AYC had an inhibitory effect on neutrophil immersion in CER+LPS-AP mice (Figure 4E).

### Emodin Inhibited AMs Pyroptosis and Secretion of Inflammatory Factors in SAP-ALI Mice

**FIGURE 5:**
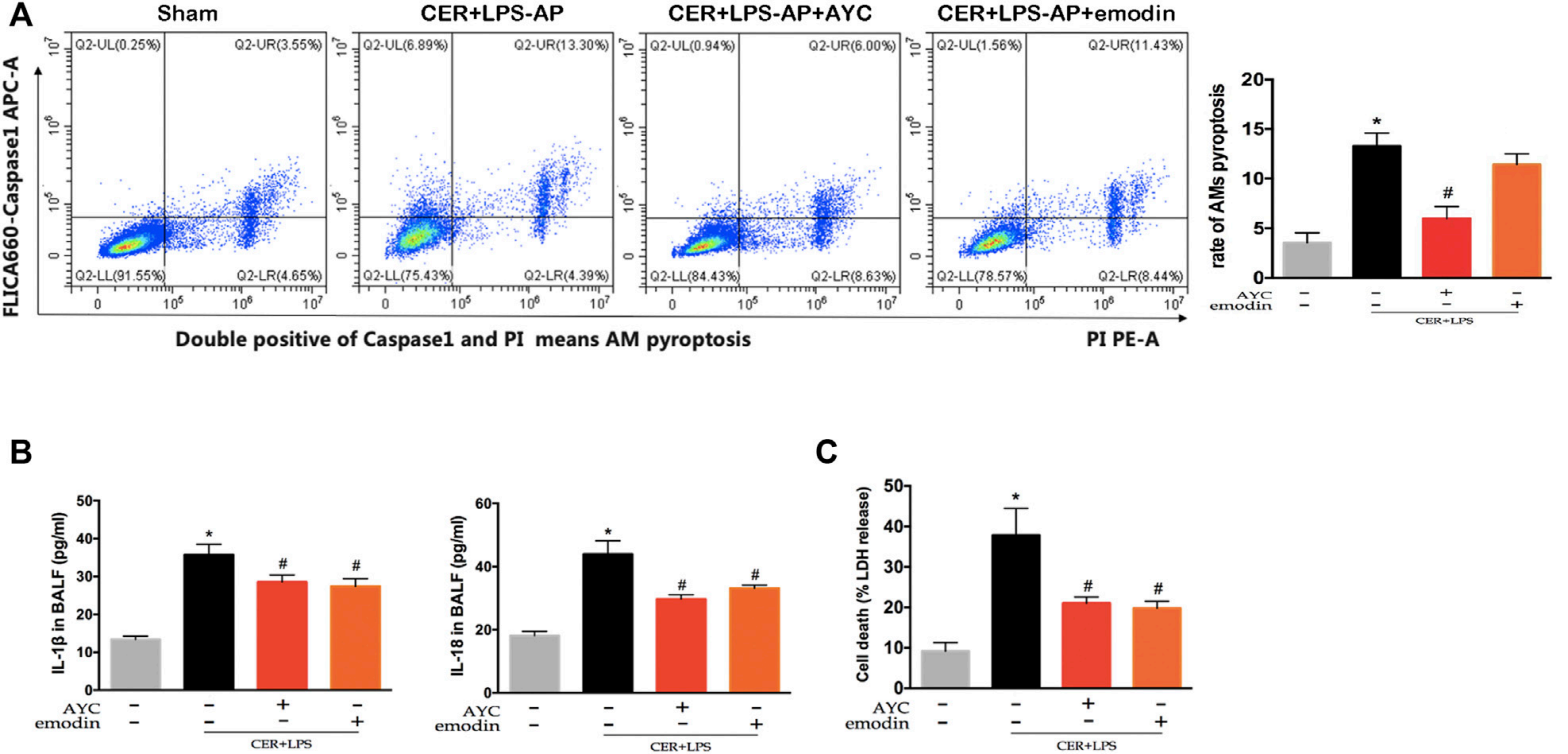
Emodin inhibited AMs pyroptosis and secretion of inflammatory factors in SAP-ALI mice. **(A)** The rate of pyroptotic AMs determined by flow cytometry from sham mice, CER+LPS-AP mice, CER+LPS-AP + AYC mice, and CER+LPS-AP + emodin mice. Double-positive staining cells (caspase-1 and PI) were regarded to be pyroptotic cells. **(B)** the levels of IL-1β and IL-18 of each group in BALF supernatant. **(C)** The level of LDH of each group in BALF supernatant. ^*^ *p* < 0.05 vs. the controls. ^#^ *p* < 0.05 vs. CER+LPS-AP mice.

Similarly, AMs from CER+LPS-AP mice displayed a higher ratio of pyroptosis compared with the sham group. Of note, either emodin or AYC could prevent the AMs pyroptosis in CER+LPS-AP mice (Figure 5A). Meanwhile, IL-1β and IL-18 levels in BALF were higher in CER+LPS-AP mice compared with those in the sham group, while levels of these cytokines decreased after emodin or AYC treatment (Figure 5B). In addition, either emodin or AYC treatment could decrease the AP-induced LDH release (Figure 5C). In short, these findings suggested that acute pancreatitis induced AMs pyroptosis in lung tissue. Meanwhile, either emodin or AYC could rescue AMs from their pyroptotic process.

### Emodin Inhibited AMs Pyroptosis by Targeting NLRP3-Caspase1-GSDMD Pathway in SAP-ALI Mice

**FIGURE 6:**
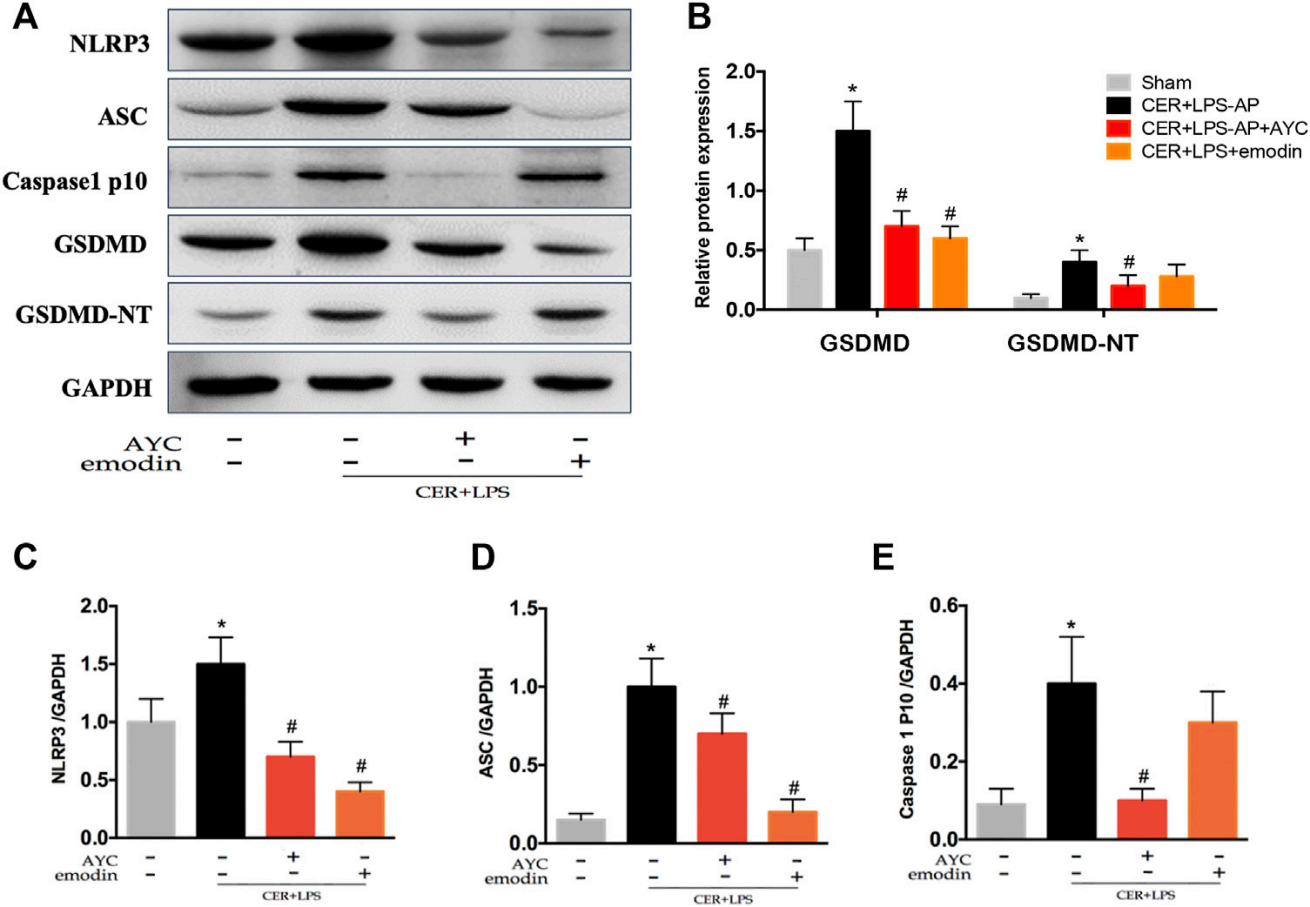
Emodin inhibited AMs pyroptosis by targeting NLRP3-Caspase1-GSDMD pathway in SAP-ALI mice. **(A–E)** Western blots and quantitative analysis of pyroptosis-related proteins (NLRP3, ASC, Caspase1 p10, GSDMD, and GSDMD-NT) relative to GAPDH from sham mice, CER+LPS-AP mice, CER+LPS-AP + AYC mice, and CER+LPS-AP + emodin mice. ^*^ *p* < 0.05 vs. the controls. ^#^ *p* < 0.05 vs. CER+LPS-AP mice.

Finally, the pyroptosis-related protein expressions were detected by western blot. The results showed that NLRP3, ASC, Caspase1 p10, GSDMD, and GSDMD-NT expressions in AMs were markedly elevated in CER+LPS-AP mice. Moreover, emodin showed the same effect as AYC, both significantly inhibiting the NLRP3, ASC, Caspase1 p10, GSDMD, GSDMD-NT protein expression in AMs (Figures 6A–E).

## Discussion

Acute lung injury (ALI) is one kind of primary or secondary disease caused by various intrapulmonary insults and extrapulmonary factors (including but not limited to sepsis, shock, pneumonia, pancreatitis etc) (Cao et al., 2016). Despite a number of methods that have been widely used for the treatment of AP-associated ALI, the mortality and morbidity still remain comparatively high with no approved effective drug or medical therapy so far. Previous research has revealed that emodin could alleviate AP-induced lung inflammatory response syndrome and acute lung injury via Nrf2/HO-1 signaling (Gao et al., 2020; Xu et al., 2021). However, systematic elucidation of the underlying therapeutic mechanism still calls for further investigation. In this study, we established two clinically relevant SAP-ALI models, including primary lung injury caused by caerulein combined with LPS (CER+LPS) and secondary lung injury induced by 3.5% sodium taurocholate (NaT). CER+LPS was an improved method for AP modeling compared with caerulein alone, since only caerulein stimulation did not cause injury of lung, liver, kidney or any other non-pancreatic organs. It has also been reported that the combination of caerulein and LPS could be used to construct severe AP models (Ding et al., 2003; Lerch and Gorelick, 2013). Besides, sodium taurocholate is commonly used to induce SAP model and mainly simulated AP model caused by bile reflux during lower common bile duct obstruction (Aho et al., 1983). As confirmed in our experiment, both sodium taurocholate and CER+LPS could cause damage to the pancreas itself as well as non-pancreatic organs (lung, liver, kidney, etc.). Based on the results, we demonstrated that emodin treatment ameliorated AP-associated lung injury and inflammatory response by inhibition of NLRP3/Caspase1/GSDMD-mediated AMs pyroptosis.

Consistent with previous findings (Gao et al., 2020; Jiang et al., 2021), we found that emodin attenuated AP-associated lung injury and mitigated excessive inflammatory response, as assessed by H&E staining, inflammatory cytokine levels and MPO activity. IL-1β and IL-18, as important promoters of inflammation, possess great potential to recruit immune cells like neutrophils and accelerate the release of other inflammatory mediators such as TNF-α and IL-6, triggering inflammation cascades to amplify the inflammatory response significantly during the course of AP (Ma et al., 2012; Bunbupha et al., 2015). MPO is regarded as a reliable marker for neutrophil activation. In this study, we found that emodin to a great extent downregulated the levels of IL-1β, IL-18, TNF-α, and MPO activity in lung tissue in both two types of SAP-ALI models, which is consistent with the protective role of emodin in reducing oxidative stress and inflammasome signals (Xia et al., 2019). In general, the interaction between pulmonary and systemic inflammation enhances the cascade of inflammation within the lungs and therefore exaggerates the progression of SAP-ALI.

Pyroptosis, a kind of caspase1-dependent inflammatory programmed cell death, is a molecular pattern leading to activation of procaspase-1 and secretion of IL-1β and IL-18 that are inherently associated with the inflammasome activation (Zeng et al., 2019). Cell death ways and activation of alveolar macrophages are considered as major factors responsible for the progression of uncontrolled pulmonary inflammation during ALI (Aggarwal et al., 2014; Niesler et al., 2014). Accumulating evidence has suggested that inflammasome-dependent AMs pyroptosis is closely related to ALI induced by a variety of challenges (e.g., lipopolysaccharide, cardiopulmonary bypass, and ischemia-reperfusion *et al*) (Wu et al., 2015; Hou et al., 2018; Fei et al., 2020). It has been reported that AMs are involved in the progression of AP from local pancreatic injury to pulmonary dysfunction, through the release of various substances like inflammatory cytokines, nitric oxide (NO), and arachidonic acid metabolites (Gea-Sorlí and Closa, 2010). Therefore, in this experiment, AMs were accordingly selected as the target cells in the treatment strategy for AP-related lung injury. Moreover, Cheng *et al* reported that a large amount of NO produced by AMs in the lung tissue might be a cause of pulmonary inflammatory damage secondary to sodium taurocholate-induced AP rats (Cheng et al., 2010). However, the specific regulatory role of AMs during SAP-ALI still remains unclarified. To identify this type of pyroptotic cells, active caspase-1 and PI positivity, downstream inflammatory factors and the LDH level were also examined. In this study, the ratio of pyroptotic AMs was increased in the diseased lung tissue, indicating AMs pyroptosis was related to ALI that was induced in NaT-AP rats and CER+LPS-AP mice. Emodin was previously reported to strongly inhibit GSDMD-mediated pyroptosis induced by myocardial ischemia/reperfusion in cardiomyocytes (Ye et al., 2019). Our findings revealed that emodin could as well exert intensive inhibition on AMs pyroptosis in two types of AP models, and its inhibitory effect was similar to that of AYC.

Activation of NLRP3 inflammasome in macrophages played critical roles in the pathogenesis during SAP-ALI (Wu et al., 2020). NLRP3 deficiency or inhibitor attenuated excessive local and systemic inflammation in experimental SAP-ALI model (Fu et al., 2018). In line with other findings, our results showed the NLRP3 inflammasome in AMs was significantly activated both in NaT-AP rats and CER+LPS-AP mice. Emerging evidence showed that emodin had an inhibitory effect on the activation of NLRP3 inflammasome in myocardial injury combined with cardiovascular dysfunction (Ye et al., 2019; Zhang et al., 2020; Dai et al., 2021). In addition, Emodin could attenuate LPS-induced ALI through regulating the NLRP3 inflammasome-dependent pyroptosis signaling pathway (Liu et al., 2021). Importantly, the administration of either emodin or AYC markedly inhibited AP-associated activation of NLRP3 inflammasome in AMs, which was supported by the downregulated expression levels of NLRP3, ASC, and Caspase1 p10. The assembly of this multimeric protein complex triggered the automatic cleavage of pro-caspase-1, which converted into caspase-1. Additionally, N-terminal GSDMD fragment (GSDMD-NT) was generated in the process after gasdermin D (GSDMD) was cleaved by active caspase-1, thus further promoting membrane pore formation and the subsequent inflammatory cascades. Initiated by activation of the inflammasome, pyroptosis did occur in AMs and aggravated ALI during the progression of acute pancreatitis. Consistent with other findings, we similarly observed that the cleavage of GSDMD was promoted under acute pancreatitis. Moreover, both emodin and AYC could downregulate the expression of GSDMD and GSDMD-NT in AMs. To sum up, all evidence favoring the therapeutic effect of emodin on NaT-AP rats and CER+LPS-AP mice was closely related to the inhibitory effect of emodin on AMs pyroptosis by targeting the NLRP3-Caspase1-GSDMD pathway.

## Conclusion

In summary, the discovery of pyroptosis has greatly broadened our understanding of the pathogenesis of ALI, and in turn targeting this manner of cell death provides new avenues for the effective treatment and management of SAP-ALI. This study used two experimental AP models to explore the roles of AMs pyroptosis during SAP-ALI and pointed out a significant correlation between emodin and AMs pyroptosis signaling pathways. In conclusion, we discovered that emodin could exhibit a therapeutic effect on SAP-ALI via regulating NLRP3-Caspase1-GSDMD signaling pathway. Accordingly, our present study may provide some alternative signaling ways for explaining the pathogenesis of SAP-ALI as well as a novel therapeutic option for treating this disease.

## Data Availability

The original contributions presented in the study are included in the article/Supplementary Material, further inquiries can be directed to the corresponding author.
