# Supplementary material for: Emodin Ameliorates Acute Pancreatitis-Associated Lung Injury Through Inhibiting the Alveolar Macrophages Pyroptosis
Source: Front Pharmacol. 2022 Jun 2;13:873053. doi: 10.3389/fphar.2022.873053 (PMC9201345; doi:10.3389/fphar.2022.873053)

Supplementary figures

R represents for rats; M represents for mice

S: sham group
AP: acute pancreatitis group (model group)
A: AYC group;
E: emodin group

Applied for all images

Figure 1S

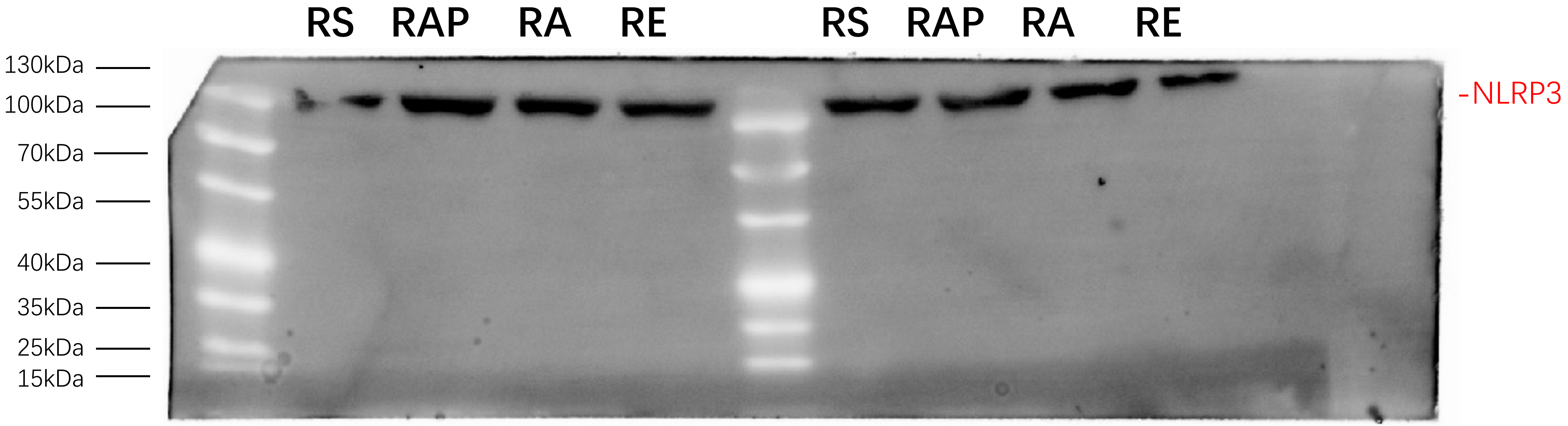

RS
RAP
RA
RE
RS
RAP
RA
RE
130kDa
100kDa
70kDa
55kDa
40kDa
35kDa
25kDa
15kDa
-NLRP3

Figure 2S

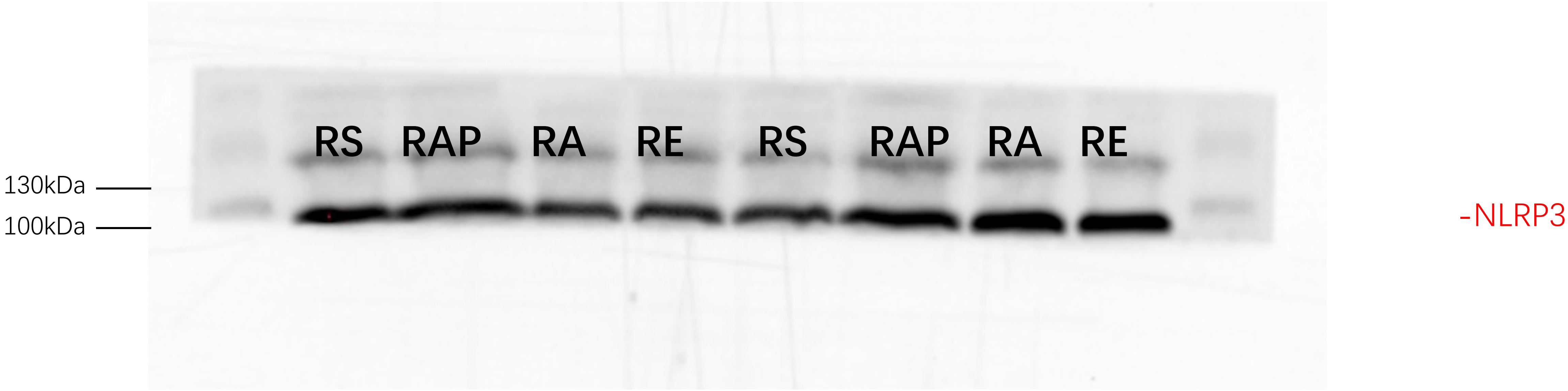

Figure 3S

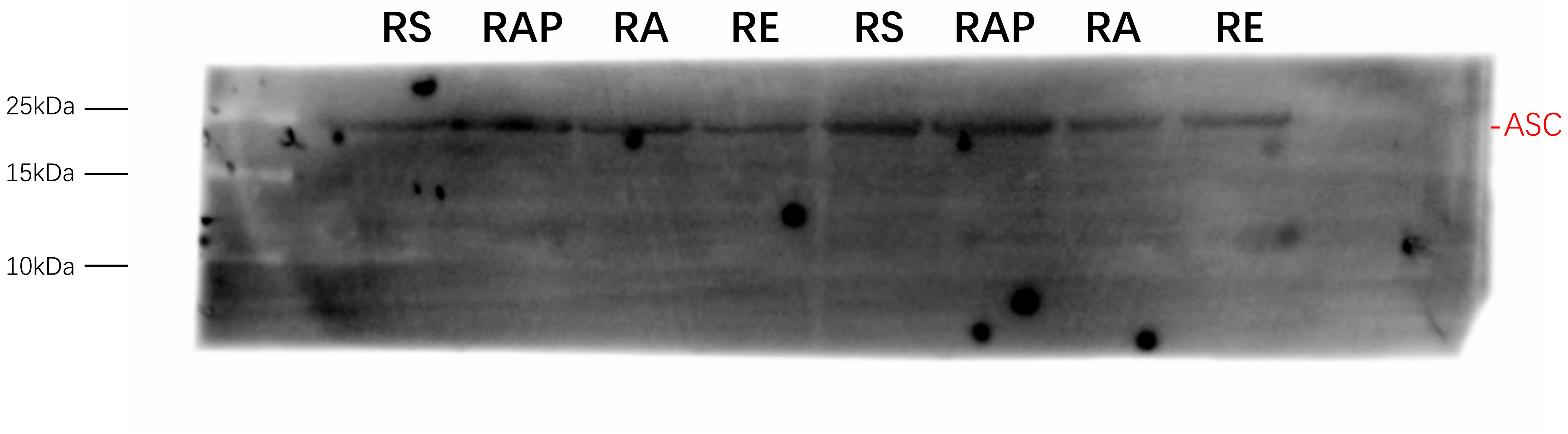

Figure 4S

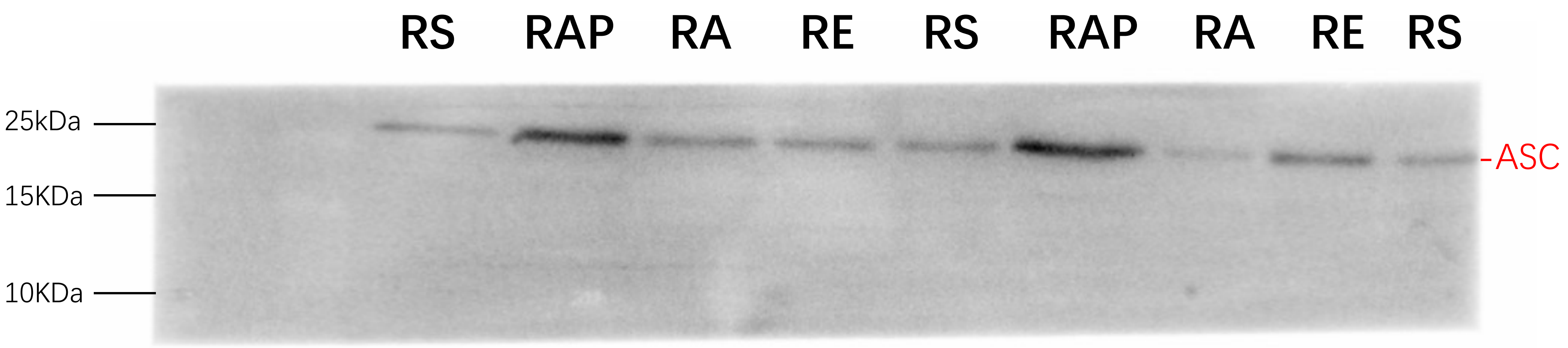

Figure 5S

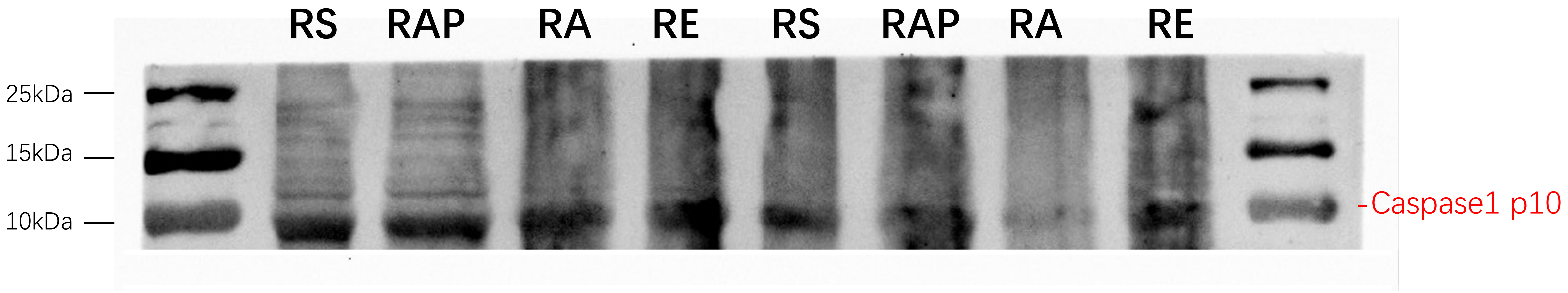

Figure 6S

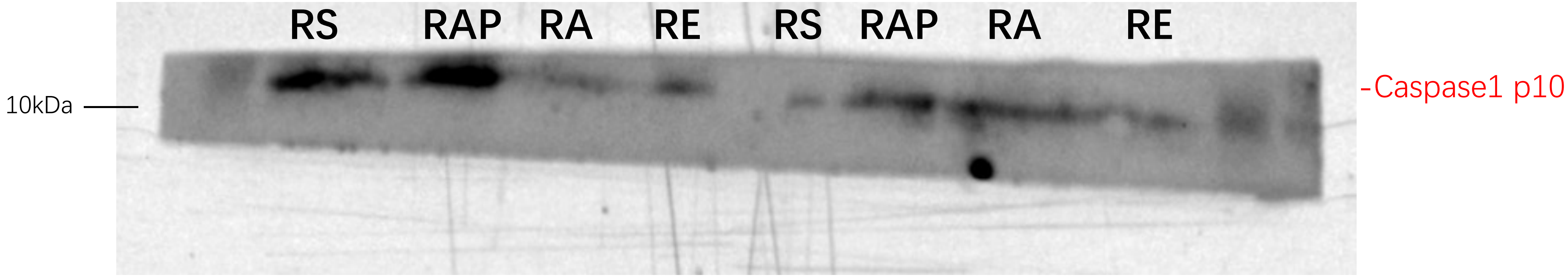

Figure 7S

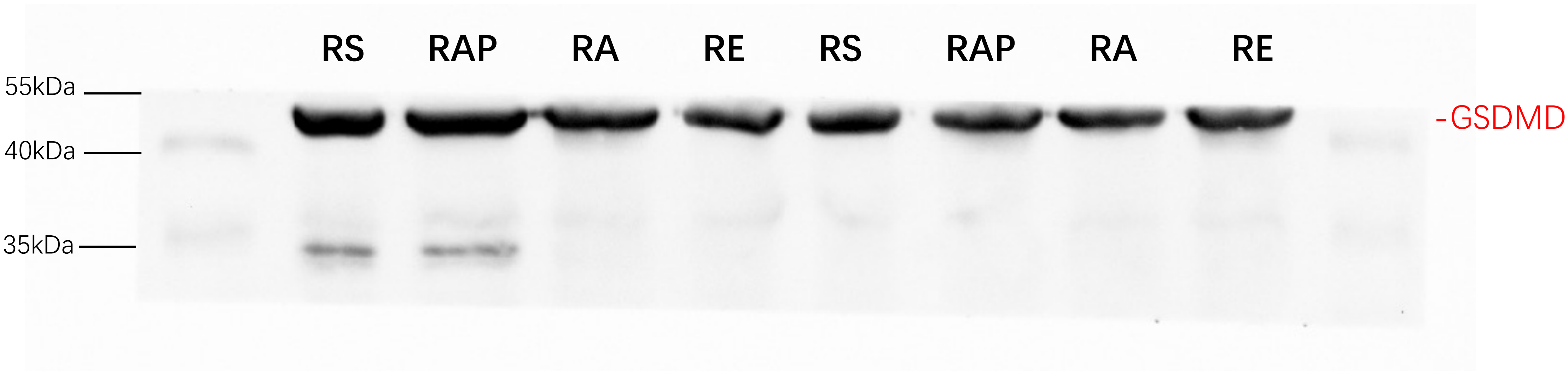

Figure 8S

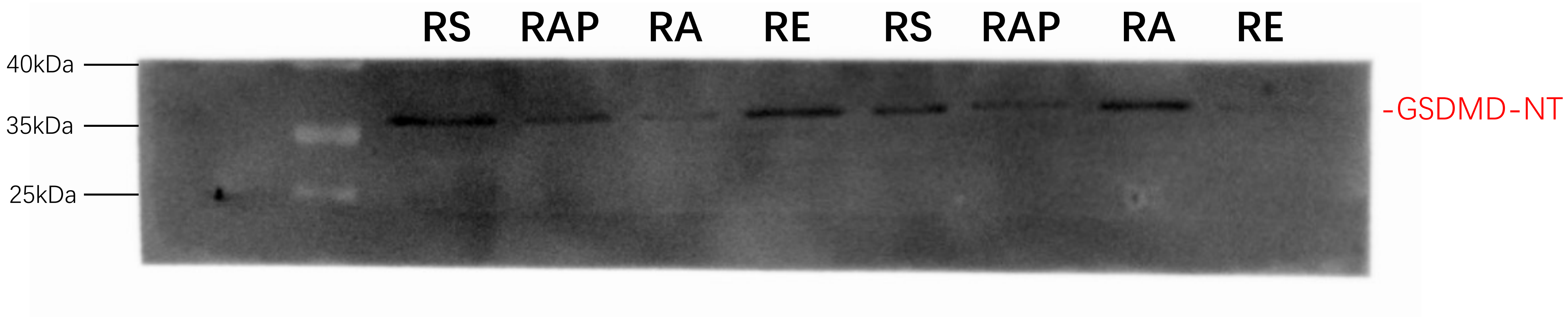

Figure 9S

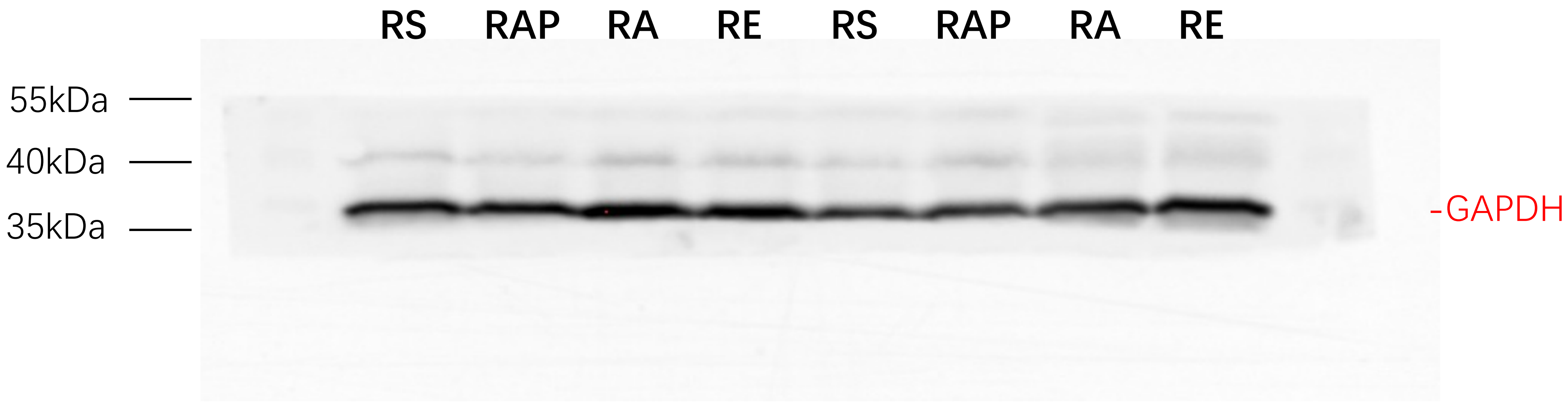

Figure 10S

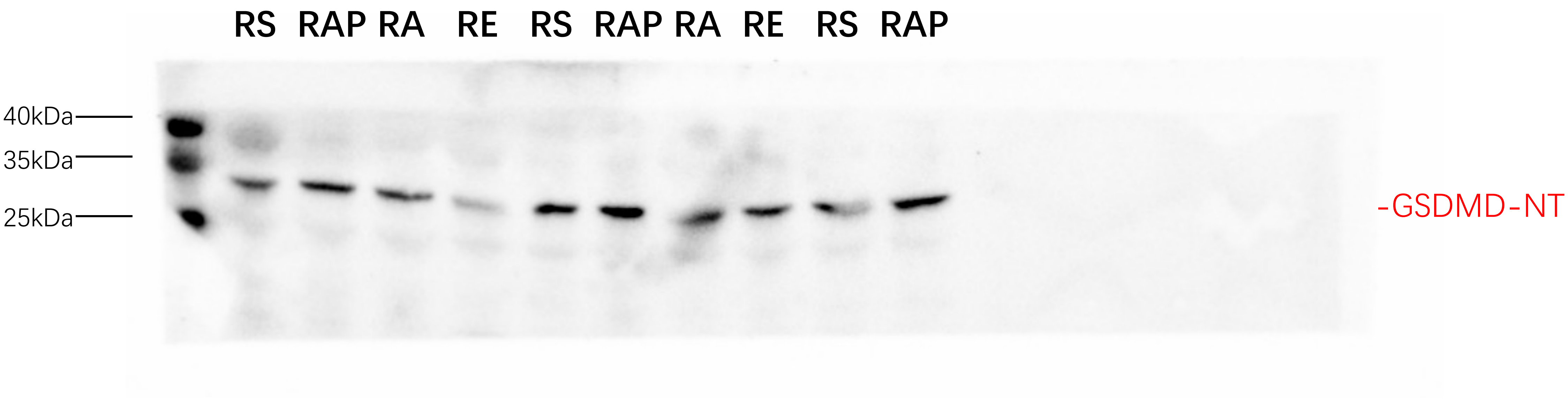

- mice

Figure 11S

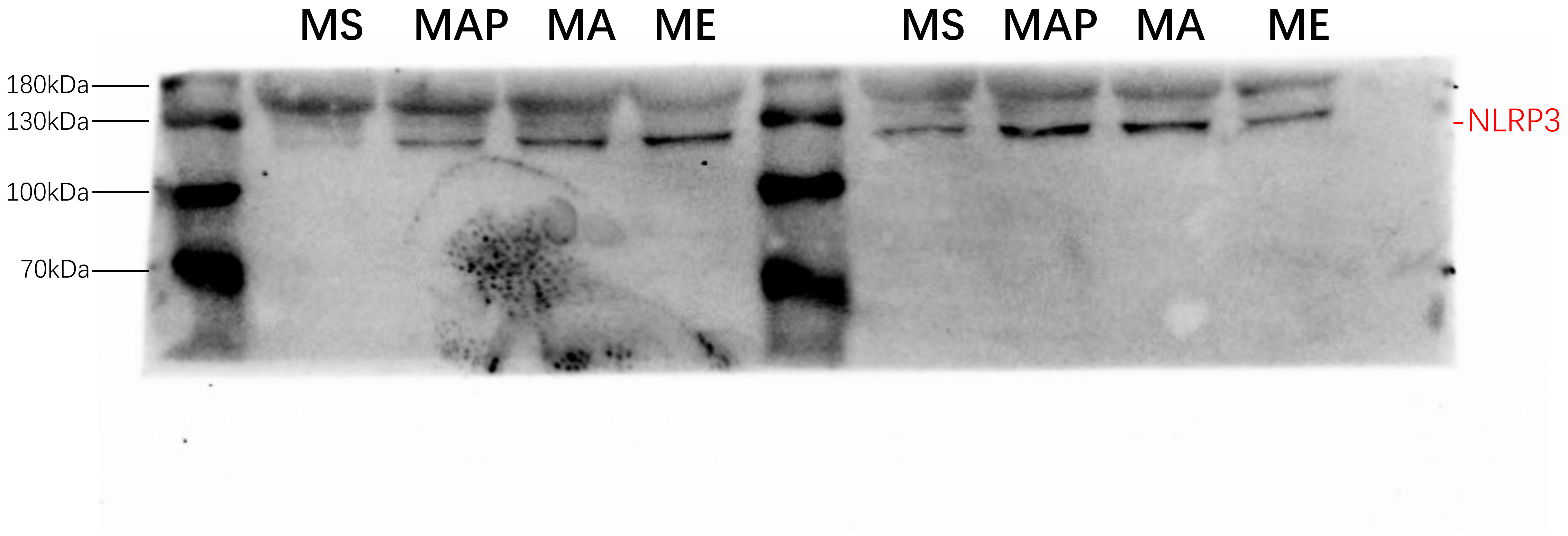

Figure 12S

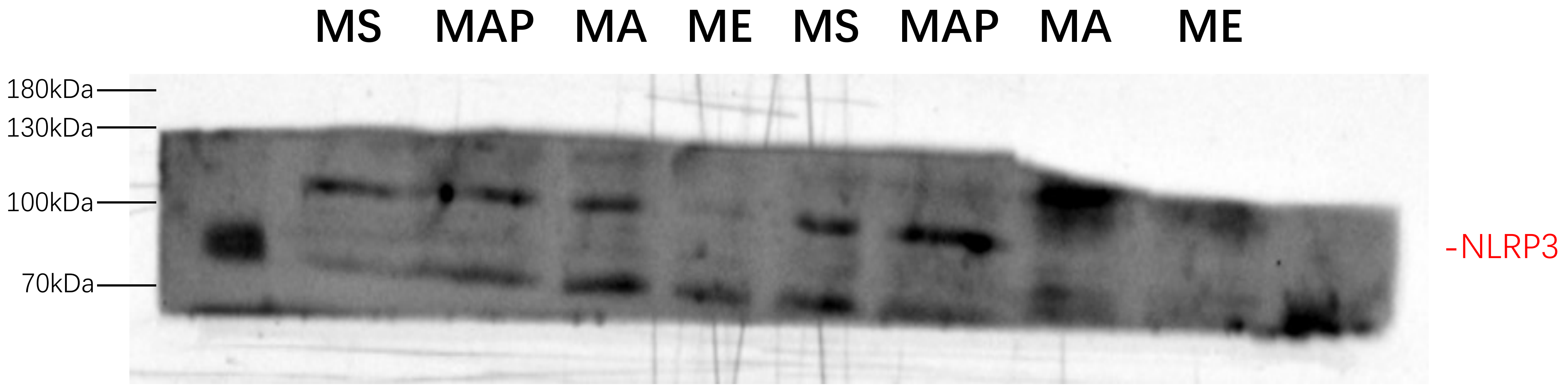

Figure 13S

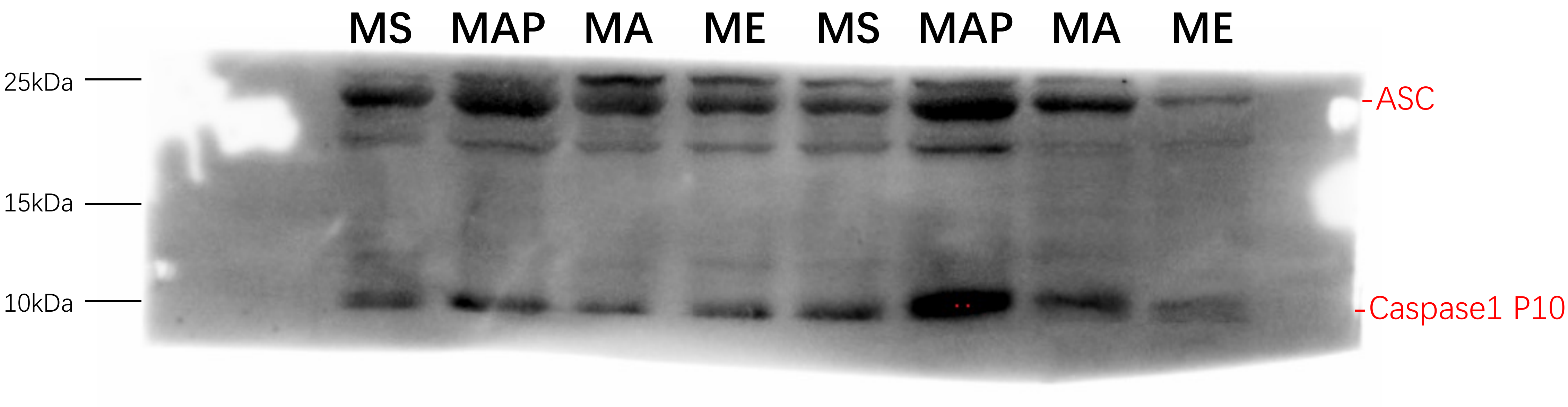

Figure 14S

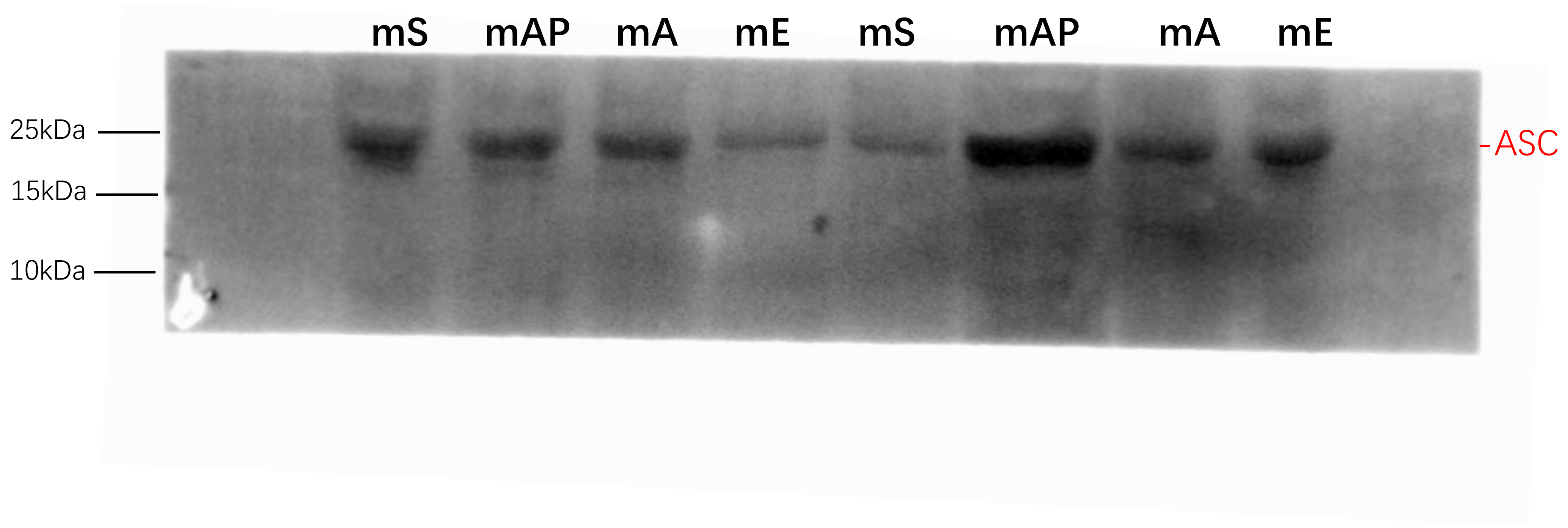

Figure 15S

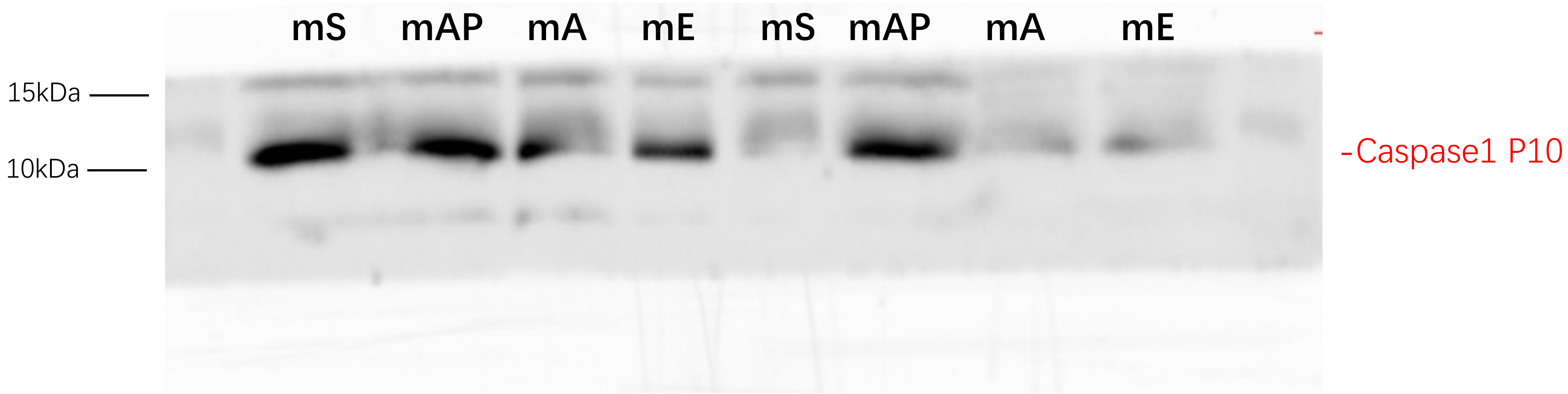

Figure 16S

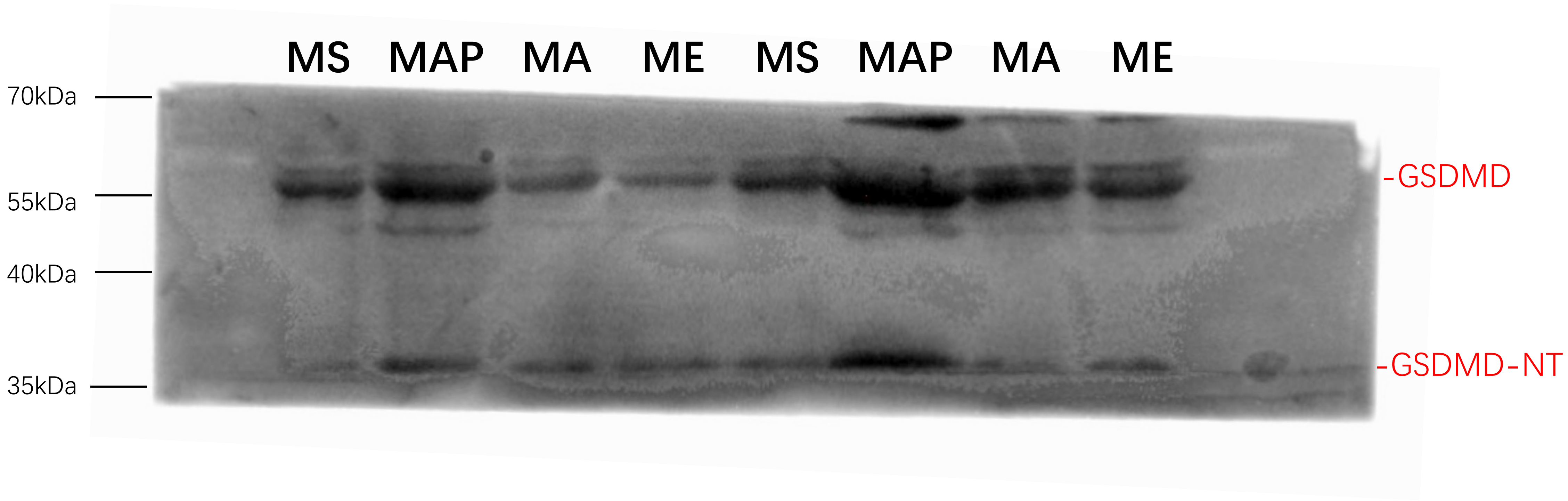

MS MAP MA ME MS MAP MA ME
70kDa
55kDa
40kDa
35kDa
-GSDMD
-GSDMD-NT

Figure 17S

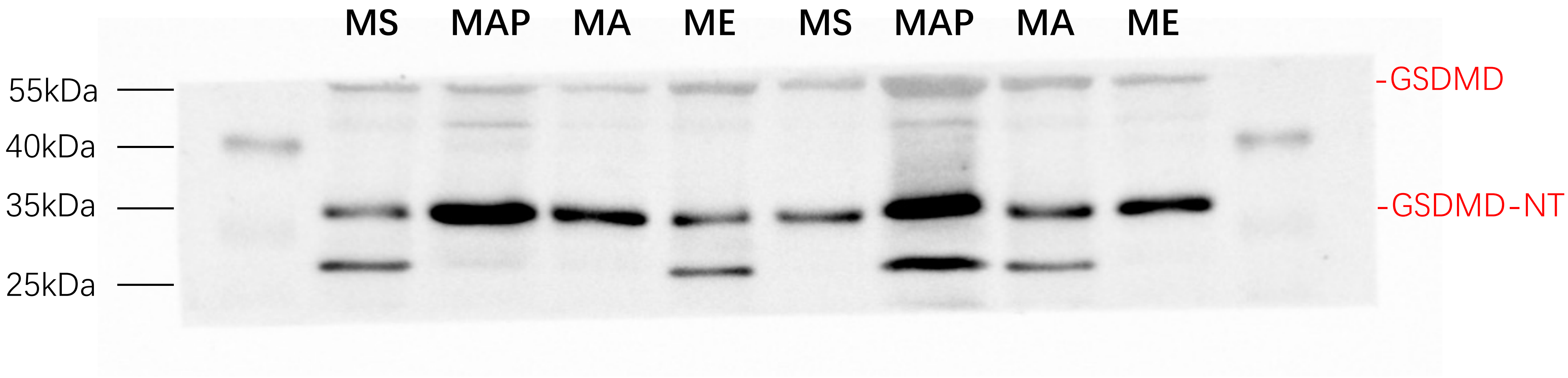

MS
MAP
MA
ME
MS
MAP
MA
ME
55kDa
40kDa
35kDa
25kDa
-GSDMD
-GSDMD-NT

Figure 18S

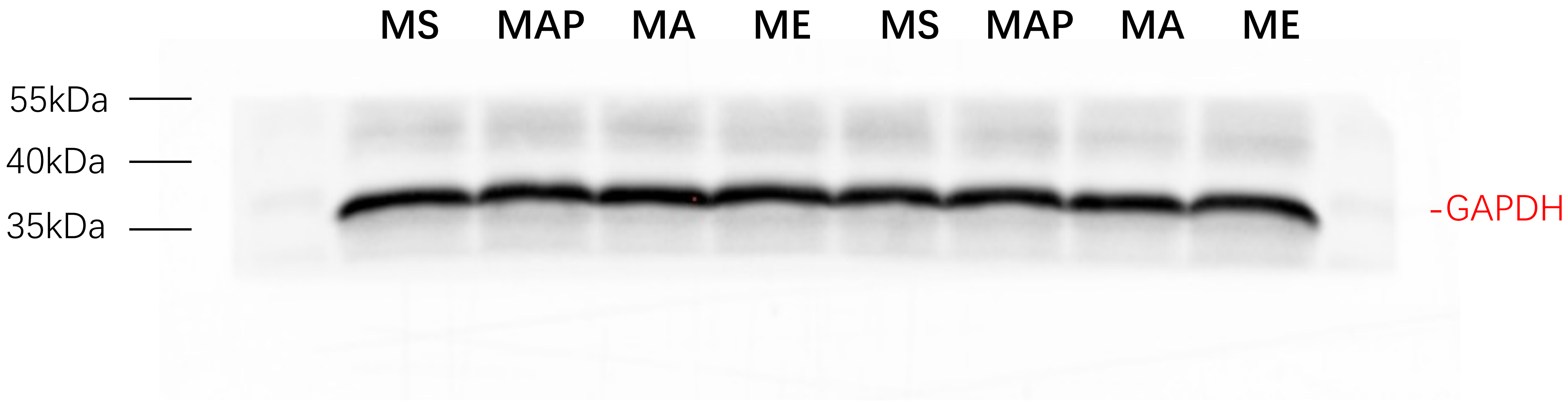

Supplement: Supplementary file 1 [file DataSheet1.pdf]
